# An Assessment of Impact of Leadership Training on Health System Performance in Selected Counties in Kenya

**DOI:** 10.3389/fpubh.2020.550796

**Published:** 2021-02-26

**Authors:** Tecla Chelagat, James Rice, Joseph Onyango, Gilbert Kokwaro

**Affiliations:** Strathmore University Business School, Institute of Healthcare Management, Nairobi, Kenya

**Keywords:** impact, health systems performance, leadership development, team coaching, Kenya

## Abstract

**Introduction:** The provision of health care services in Kenya was devolved from the national government to the counties in 2013. Evidence suggests that health system performance in Kenya remains poor. The main issue is poor leadership resulting in poor health system performance. However, most training in Kenya focuses on “leaders” (individual) development as opposed to “leadership” training (development of groups from an organization). The purpose of that study was to explore the impact of leadership training on health system performance in selected counties in Kenya.

**Methods:** A quasi-experimental time-series design was employed. Pretest, posttest control-group design was utilized to find out whether the leadership development program positively contributed to the improvement of health system performance indicators compared with the non-trained managers. Questionnaires were administered to 31 trained health managers from the public, private for-profit, and private not-for-profit health institutions within the same counties.

**Results:** The pretest and posttest means for all the six health system (HS) pillar indicators of the treatment group were higher than those of the control group. The regression method to estimate the DID structural model used to calculate the “fact” and “counterfactual” revealed that training had a positive impact on the intended outcome on the service delivery, information, leadership and governance, human resources, finance, and medical products with impact value ≥1 (57.2).

**Conclusion:** The study findings support both hypotheses that trained health care management teams had a significant difference in the implementation status of priority projects and, hence, had a significant impact on health system performance indicators compared with non-trained managers.

## Introduction

Despite the significant investment of about $8 trillion in global health care spending, millions of people, especially from the developing countries, still die each year from preventable causes (1). This has been attributed, among other factors, to the fact that a majority of the people responsible for leading, managing, and governing health care have little or no preparation to succeed in this role (2). Leadership and governance form one of the critical building blocks of the health system (3) and is now recognized as an essential determinant of strengthening national health systems and therefore at the core of achieving health-related goals (4, 5). Good leadership is an enabler of good governance, management, service delivery, and overall improvement of population health (6). Thus, when people who govern, managers, service providers, patients, and community members consistently practice good leadership, this will ultimately result in a healthier population (7–9).

### Overview of Global Health System

A health system is described as “all of the organizations, institutions, resources, and the people whose primary purpose is to improve health” (10). The health system is, therefore, a means to deliver effective and affordable care toward meeting health goals (11). To achieve Sustainable Development Goals, health system strengthening strategies (such as inspired leadership, sound management, and transparent governance) are critical as they can catalyze expanded investments in health (12). Strengthening health system, therefore, means addressing key constraints in each of these areas, with a goal of promoting effective access, improved quality, and increased utilization of health services (10). This includes developing practical approaches for monitoring and evaluating the various levels of system inputs, processes, outputs, and outcomes (13). That is why creative education is required for health workers to drive the improvement of health system performance.

The World Health Organization and global partners have developed a framework for measuring health system performance that is composed of six core components or “building blocks” (3). The two pillars (leadership and health information systems) are components that crosscut to form the basis for the overall policy and directive of the other health system building blocks. The leadership and governance pillar is concerned with procedures that promote commitment and accountability. Financing and the workforce are key input components, while medical products/technologies and the provision of services represent immediate system output. The framework provides a structure for this complex system by defining these elements, allowing the identification of measures and measurement methods for monitoring and evaluation (10).

Sustainable Development Goals 3 (health) and 4 (education) are linked to health system strengthening. These goals aim at producing competent health workers and also research results that inform health policies and practices (14). A majority of health managers in many developed countries, including Kenya, are qualified health professionals who may have the technical skills but who are not skilled or experienced in management and leadership before being offered a managerial role (15). The International Hospital Federation (IHF) lists leadership among the five competencies that modern health care managers need to have (16). It is therefore expected of health managers to acquire these skills through additional in-service training (17). The newly qualified health workers are usually posted based purely on clinical skills. Most of these health workers are ill-prepared for leadership and managerial duties. They are often expected to undertake new positions without these skills, contributing to an even more broken health system (7). A broken health system results in more illness and death, irrespective of the existence of the public health and medical knowledge to significantly reduce illness and save millions of lives every year, especially in developing countries (18). Consequently, there has been a surge of leadership training in the past decade due to its high association with increased positive changes in service delivery and its health outcomes (3).

### The Kenya Health Policy and the National Development Agenda

The Fourth Kenya Health Sector Strategic and Investment Plan, 2014–2018 (KHSSP IV) was developed to align with Kenya's devolved system of governance (19, 20). A mid-term review of the health sector strategic plan under the leadership and governance performance reported positive adoption of the Kenya Health Policy, 2014–2030. The aim was to provide guidance on the creation of health sector plans and the effective creation of county health strategic plans during their first year of implementation (21). Though this was the case, the study highlighted key governance and leadership challenges. The challenges include (a) the lack of structured guidelines for creating a county health management agency framework to enhance health goals and better coordination, (b) the lack of a cooperation structure to encourage and organize international assistance better and to foster transparency, and (c) the lack of a focused capacity building program by the Ministry of Health for the counties (22). Given the highlighted challenges, the Kenya Health Policy, 2014–2030 offers a system that can leverage and align health care services across the devolved system, with the national government providing overall policy direction, strategic leadership, and stewardship aimed at defining the strategic vision of the health agenda in Kenya (22). The successful implementation of the policy is, therefore, dependent on the collaborative efforts and synergies of all the stakeholders and actors through the establishment of an effective partnership framework via new institutional and management arrangements. Health leadership and governance is one of Kenya's health policy orientations and aspiration toward the delivery of the health agenda. Health care leadership and governance, therefore, relates to how the oversight of the delivery of health and related services is provided. The Kenya Health Policy Framework illustrates how leadership, management, and governance training are connected to improved human resources for health competencies, resulting in improved health system performance indicators for better health in a responsive manner.

### Leadership Development Interventions

Literature regarding health system leadership suggests that there are potential benefits of health care leadership preparation for the health workforce. Systematic inquiries on leadership development strategies and their application, however, are minimal (23–26). Hence, the need to develop robust and sustainable health systems therefore needs innovation, like groundbreaking preparation for the health workforce (10). Based on the above evidence, comprehending the effective implementation of leadership development for health is vital (27). Leadership is commonly regarded as the key to effective health care systems (28). However, most of the training in Kenya focuses on “leaders” development (individual) as opposed to “leadership” training (group growth from an organization). The previous strategy had no effect. Several partnering agencies and institutions have been designing and implementing leadership training across the counties as informed by the Management Sciences for Health (29), Kenya's Ministry of Health and Funzo Kenya (30) training needs assessment for health care managers, and the health sector strategic investment plan for the year 2013 (30, 31). The Strathmore leadership development program was proposed as a response to the Ministry of Health and its partner's training needs report. The curriculum was designed to provide an opportunity for the teams to practice knowledge, skills, and attitude to address real workplace policy and systems challenges to produce measurable results toward improving health performance. The Leading High-Performing Healthcare Organizations (LeHHO) program was proposed as a response to the Ministry of Health and its partner's training needs report. The curriculum was designed to provide an opportunity for the teams to practice knowledge, skills, and attitude to address real workplace policy and systems challenges to produce measurable results toward improving health performance. Taking into account that the program evaluation activities for the LeHHO program could be described as primarily formative or process-oriented, little is known of the training-attributed outcome and impacts.

### Aim of the Study

The aim of the study was to assess the effectiveness and possible effect of leadership development training on the performance of Kenya's county health care system, through the implementation of institutional improvement priority projects. We explored the contribution of the application of leadership practices and team coaching through institutional implementation projects toward strengthening health system performance. Building on our earlier study (15) and our most recent study on “Effect of project-based experiential learning on the health service delivery indicators: a quasi-experiment study” (32), the current study focused on the entire health system pillars (service delivery, information, leadership and governance, human resources, finance, and medical products). In addition to the findings of two earlier mentioned studies, our study explored the relationships between the six health system pillars, within the treatment, and between the treatment and control group. The program is challenge-driven, and it integrates team coaching and challenge model in its training design.

### Research Questions

The aim of the study was to assess the effectiveness and impact of leadership development training on the health system performance through the implementation of institutional improvement priority projects. To realize the stated purpose, we posed the following questions.

Q1: What is the implementation status of institutional improvement projects post-leadership development training between the years 2011 and 2016 for both the trained and non-trained managers?
Q2: What is the impact of the priority challenge projects implemented on the prioritized health system performance indicators?

### Hypotheses

H1: The trained health care management teams had a significant difference in the implementation of institutional improvement priority project post-training as a contribution of leadership development program compared with non-trained managers.
H2: Operationalization of governance and leadership practices by implementing institutional priority projects by trained managers had a positive impact on health system performance indicators compared with non-trained managers.

## Methodology

The quasi-experimental time-series design was utilized to establish a cause–effect relationship between variables as recommended by Neuman (33). Experiment design is a procedure that aids the investigator to control variations of the independent variables correlated with the changes in the independent variables (34). Quasi-experiments are considered effectual for the reason that they use “pre–post testing.” The tests are therefore done prior; any data are collected to ascertain any confounds or if any participants have certain predispositions (35). Quasi-experiments, therefore, have already existing independent variables, including baseline indicators for this research (36, 37). Quasi-experiment was suitable for this study because they are extremely valuable when true experiment such as randomized control trial is not feasible, and when appraising the effect of policy changes or educational interventions (36). Given the context of the training, a quasi-experimental design is easier to build than real experimental designs. Since quasi-experimentation is natural experimentation, results in one can be generalized to other subjects and environments, making it possible to make some generalizations about the population (37). Hypothesis testing of causal relationships was done with consideration of the leadership development training as a predictor variable for possible change and priority project indicators outcomes as an effect. Semi-structured questionnaires were administered to measure the health system performance indicators of interest and compared with the non-treatment group within the same time span to test for statistical significance difference as an effective leadership intervention program. Pretest–posttest control-group design was utilized to find out whether the “LeHHO” program positively contributed to improving the efficiency of health care programs to the improvement of health system performance indicators compared with non-trained managers.

### The LeHHO Program Delivery Approach

Strathmore University Business School has developed and implemented a Health Managers' Leadership training program based on the competency domains upon which this study is based. It is called the “Leading High-performing Healthcare Organizations” (LeHHO). The program was developed and first implemented in the year 2010 in partnership with Management Sciences for Health (MSH) and Ministry of Health (MOH). The program was funded for 5 years by the United States Agency for International Development (USAID). The program aimed to empower national and county health management teams in Kenya to improve health service delivery after devolution (32). The program has been implemented in nine cycles between the years 2010 and 2016 and trained over 200 health care managers and leaders (32). A vital aspect of leadership development training was the integration of facility improvement projects and team coaching in the curriculum (32). The idea is that it is through the implementation of such projects that participants can translate leadership training theory into practice and have a positive impact on institutional health system performance. The role of the team coach, therefore, was to help teams demonstrate their own leadership skills through practice by clarifying the project's objective, holding the teams accountable, monitoring the project's progress, and participating in experience sharing workshop. These workshops were embedded in the five modules and the project's teams were expected to present their progress after every module break. This study covers the period from 2010 to 2016. During this period, LeHHO trainees identified and implemented 69 projects spread across 39 health facilities and 19 counties in Kenya. To this end, there is an information gap on (a) whether the teams transferred the knowledge through the implementation of the priority projects selected to the real work environment and (b) whether there are impacts on the intended outcomes. These are the research gaps that our current study intended to fill.

### Population and Sampling

The target population is composed of senior health care management teams drawn from 19 counties in Kenya who had undergone the LeHHO program, with matching comparison health institutions from the same counties representing the public, private for-profit, and private not-for-profit health facilities whose management teams had not undergone the training. The counties represented different health system performance challenges in terms of health care workers' distribution, resource allocation, numbers and types of facilities, and their distribution and population demographics. The study sample was non-randomly drawn from the LeHHO program alumni for the years 2011–2016 at Strathmore Business School 39 project teams.

### Matching the Intervention and Comparisons Teams

The trained teams were self-selected based on interest in participating in the LeHHO intervention. Due to the absence of random assignment, intervention health facilities were matched with comparison health facilities. The matching of health facilities was done retrospectively by an independent research manager who did not have prior knowledge to the intervention. The intervention facilities were non-randomly matched with health facilities within the same County. The matching used a multiple-criteria approach including the type of facility, using categories established by the government, geography physical location, and facility size. Supplementary matching criteria were type of facility, using categories established by the government HMIS starting from referral hospitals. Even though the matching of private and faith-based health facilities adhered to the laid criteria, there was a challenge in matching the public health facilities due to the complexity in the organization of health services in Kenya under a devolved system. Out of the 39 priority projects, we were able to match 31 projects against 29 comparison facilities. The remaining eight projects were projects undertaken by national government teams, including the regulatory bodies; hence, unmatchable therefore were filtered out during impact analysis. The collected data were very vital in comparing whether there was a notable significant improvement in the selected indicators not only within the same institution over time but also when compared with other health facilities. The justification for this matching is that both facilities function under the same county health system and are governed by a similar strategic plan (32).

### Research Outcomes and Procedures

Implementation of priority projects by project teams and health system performance indicators were the two primary outcome variables of interest. It was envisioned that the effectiveness of the program in achieving its intended purpose would be reflected on the implementation status of the priority projects selected and demonstrates a positive indicator score as compared to the non-trained health care managers. Approval to carry out the research was obtained from the relevant research ethical review bodies to guarantee the integrity of the study and data collection. The objective was to study participants with disclosure of any direct or indirect benefits and risks involved. All persons who took part in the study have received and obtained informed consent. They also were informed that their participation was voluntary and that they had the right to withdraw when they wished to without facing any penalty. Confidentiality was assured and no identifiable data or information will be released to anyone. For the intervention group, baseline data were collected during their registration to the program, endline data were collected at the end of the 9-month training, and the posttest was done between August and October 2018. The study utilized both primary and secondary data. Primary data were collected using a questionnaire, in-depth interview guide, and the project's challenge model as the observation checklist. The questionnaires are composed of closed-ended questions that sought to provide a more structured response as a snapshot of the priority projects' tangible outcome (Were the priority projects implemented? If yes, what are the impacts on health system performance indicators addressed?). Data for control teams were collected by the principal investigator with assistance from the national and county health management information systems' officers; confirmation was obtained from the health facilities by the research assistant.

### Data Analysis

The completed questionnaires were first edited for completeness and consistency and then captured electronically, and the quantitative information collected was entered into SPSS 20. A two-step statistical analysis was performed using descriptive statistics analysis, linear regression, and *t*-test, and impact was calculated using means and mean differences. Descriptive statistics analysis such as means, standard deviations, and frequencies were derived from the baseline, endline, and posttest measure of the priority project indicators for the treatment and control group. To estimate the impact of the training, the following stepwise calculation will be undertaken: (a) means and mean differences and (b) use of regression method to estimate difference-in-difference (DID) structural model below by Krueger and Card (38) and Larson and Hutchinson (39). Measuring impact using means and mean differences, in other words, the impact is the difference in mean differences (DiD). Initially, we had posited that participation in the leadership development training program would be positively associated with the attainment of priority project goals. An independent sample *t*-test was performed to test the hypothesis by comparing the means in the two groups at the baseline and endline. Linear regression was used to test the second hypothesis on impact.

## Results

### Respondent Demographics

The study findings indicated that 18 (58%) were female. The age ranged between 46 and 55 years old. Over ½ (58%) of the participants had a master's degree. From the project team leaders who participated in this study, 14 (45%) were from public hospitals, 13 (42%) were from faith-based health facilities, and 4 (13%) were from private hospitals.

### Classification and Implementation Status of Priority Challenge Projects

The current study objective aimed to evaluate the effectiveness of the leadership training on health system performance indicators from the implemented priority projects. The World Health Organization (WHO) framework for measuring health system performance was used to identify the indicators and measurement strategies for monitoring and evaluating the priority projects implemented. Table 1 provides concise statistics for the six main elements (“building blocks”) of a well-functioning health care system (leadership and governance, health information systems, finance, human resources, medical products/technologies, and service delivery). A total of 31 projects aligned to their strategic plans were prioritized by the teams. We clustered the projects according to the WHO health system building blocks (10) for analysis. Out of the 31 projects implemented, 29 (93.5%) teams achieved their desired observable outcomes. Service delivery was the most chosen challenge (research) area by the public, faith-based, and private sector, and human resources, finance, and medical products were the least chosen challenge areas. Service delivery had the highest score of 45%; health information was 23% of the projects; leadership, management, and governance (LMG) was 19%; human resources was 6%; medical products was 3%; and health finance was 3%.

**Table 1:** Challenge projects category and implementation status.

	**Health sector**				
**Health system pillar**	**No and % (project area)**	**Public**	**Faith-based**	**Private**	**DMR achieved**
Service delivery	14 (45%)	8 (57%)	4 (31%)	2 (50%)	13 (92.3%)
Information	7(23%)	4(29%)	2 (15%)	1 (25%)	7 (100%)
LMG	6(19%)	1 (7%)	4 (31%)	1 (25%)	6 (100%)
Human resources	2(6%)	_	2 (15%)	_	1 (50%)
Finance	1 (3%)	_	1 (8%)	_	1 (100%)
Medical products	1 (3%)	1 (7%)	_	_	1 (100%)
Total	31 (100%)	14 (100%)	13 (100%)	4 (100%)	29 (93.5%)

*Source: Survey data 2018*.

Table 2 presents the pretest and posttest means for health system performance indicators for all six health system (HS) pillars of the experimental group, which was higher than those of the control group. These findings present the differences between trained and non-trained manager pre-training. The highest pretest score of the treatment group was service delivery (M = 82.32, SD = 89.20) and the lowest mean was for the medical products (0.00). The highest pretest score for the control group was service delivery as well (M = 50.36, SD = 75.17), whereas the lowest score was for human resources, finance, and medical products (M = 0.00). The posttest scores for both treatment and control groups indicated a significant difference. In summary, the highest posttest for the treatment group was service delivery (M = 122.04, SD = 117.97), with human resources scored as the lowest (M = 62.5, SD = 53.03).

**Table 2:** Pretest and posttest means and standard deviation for treatment and control groups.

	**Treatment group**				**Control group**			
	**Pretest**		**Posttest**		**Pretest**		**Posttest**	
**Variables**	**Mean**	**SD**	**Mean**	**SD**	**Mean**	**SD**	**Mean**	**SD**
Service delivery	82.32	89.20	122.04	117.97	50.36	75.17	62.14	104.84
LMG	12.33	30.21	78.33	34.88	8.33	20.41	8.33	20.41
Human resources	10.00	14.14	62.50	53.03	0.00	0.00	0.00	0.00
Finance	68.00		78.00		0.00		0.00	
Information	11.71	30.11	98.57	3.78	14.29	37.80	14.29	37.80
Medical products	0.00		100.00		0.00		0.00	

*Source: Survey data 2018*.

**Figure 1:**
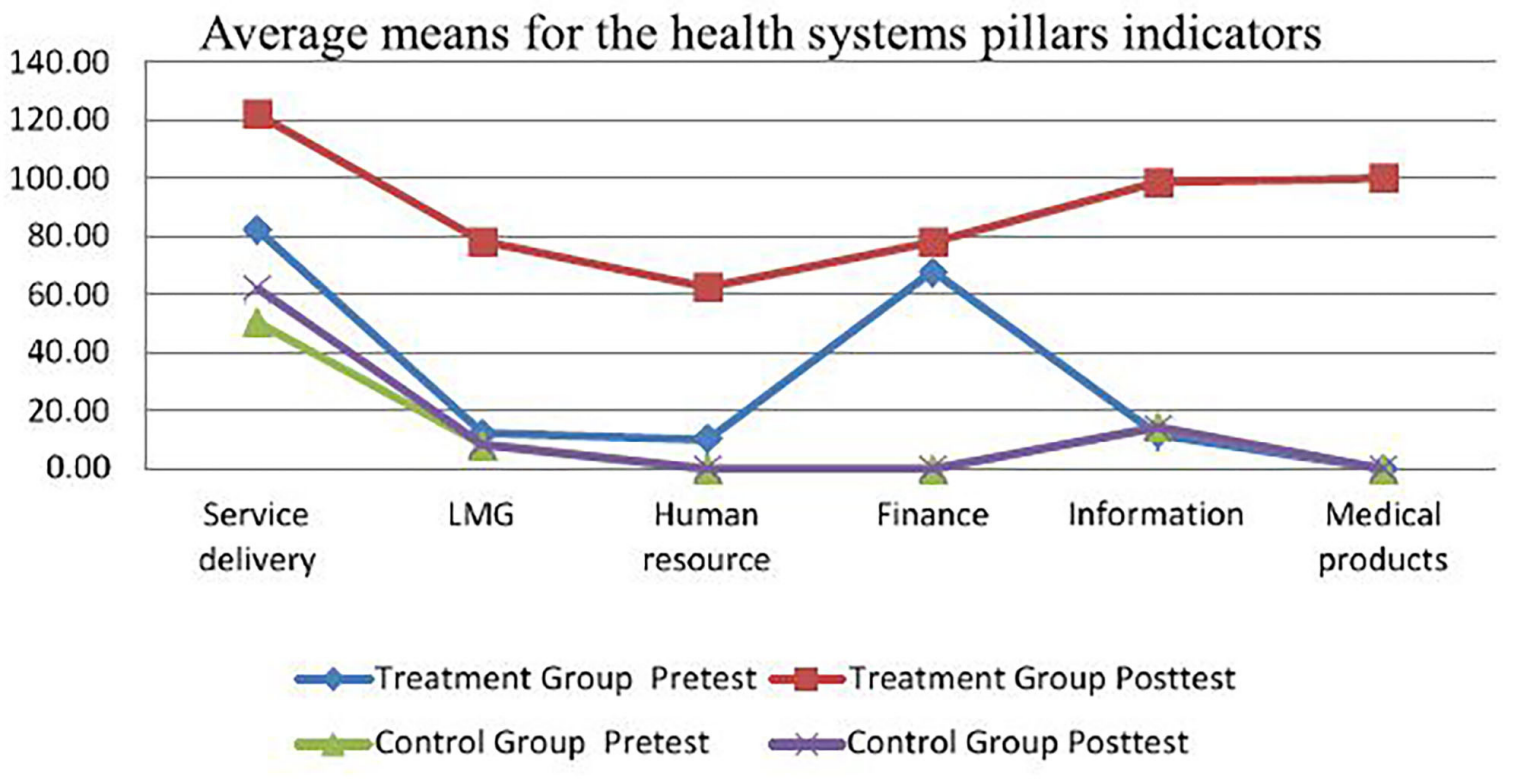
Average means per health system pillar indicator. Source: Survey data 2018.

The means for the pretest and posttest for the six health system pillars' performance indicators are graphically summarized in Figure 1.

### Results of *t*-Test

Table 3 illustrates the summary results of before and the leadership training program within pretest and posttest scores for the two groups. The pretest scores between two groups indicate that health system performance indicators for five out of six HS pillars from the experimental (trained) group were pointedly different for the control group prior to the training. The training had a positive effect on success metrics for HS with posttest performance indicators for three pillars: service delivery, leadership, and governance, and information showed substantial variances between the two groups (*p* < 0.05). The *t*-test for dependent samples indicated a difference between pretest and posttest results for service delivery within the experimental group and the control group with a confidence interval of less than .10 (*p* < 0.10). However, there was no significant difference between pretest and posttest scores for service delivery of the control group.

**Table 3:** *t*-test results.

	**Treatment**		**Control**		**Pretest**		**Posttest**	
	***t*-Stat**	***P*-value**	***t*-Stat**	***P*-value**	***t*-Stat**	***P*-value**	***t*-Stat**	***P*-value**
***Variable***	***t test (dependent samples)***				***t test (independent samples)***			
Service delivery	21.2787	0.0035	8.2714	0.0926	55.3436	0.1983	46.2807	0.0257
LMG	1.1516	0.0277	–		5.7600	0.9362	3.1174	0.0216
Human resources	(0.6913)	0.1797			(0.5303)	0.4386	(0.5303)	0.1213
Information	2.4047	0.0180			9.1609	0.4822	5.7600	0.0202
Finance	–	–	–	–	–	–	–	–
Medical products	–	–	–	–	–	–	–	–

*Source: Survey data 2018*.

### Linear Regression

Two models of regression analysis were used to test whether the leadership development training positively influenced the health systems' performance outcome of the implemented projects. The first model was intended to solely examine the impact of the training (independent variable) on health system performance indicators (dependant variable). Table 4 depicts the regression results of model 1.

**Table 4:** Regression statistics.

Multiple (*R*)	0.41					
*R*^2^	0.16					
Adjusted *R*^2^	0.15					
Standard Error	79.4285					
Observations	62					
**ANOVA**	***df***	***SS***	***MS***	***F statistic***	***F significance***	
Regression	1	74,660.5202	74,660.5202	11.83	0.0011	
Residual	60	378,533.0484	6,308.8841			
Total	61	453,193.5685				
	***Coefficients***	***Standard error***	***t-Statistic***	***P-value***	***Lower 95%***	***Upper 95%***
Intercept	32.9032	14.2658	2.31	0.025	4.3674	61.4390
Training (*X*_1_)	69.4032	20.1749	3.44	0.001	29.0475	109.7589

*Source: Survey data 2018*.

The general equation of the model will be a multiple regression as stated below:

$$\begin{array}{l} \gamma =c+B_{1}X_{1}+e_{t}\\ \gamma =\text{improved health system performance indicators}\\ \left(\text{dependent variable}\right)\\ X_{1}=\text{1 for trained and 0 for the non-trained}\\ \left(\text{independent variable}\right)\\ c=\text{constant}\\ B_{1}=\text{regression coefficient}\\ e_{t}=\text{error term} \end{array}$$

*F* statistic (*p* < 0.05) was used to estimate the accuracy of model 1 by analysis of variance. The *F* significance (0.001) is < 0.05, confirming that model 1 is significant. Also, we tested the *p*-value for coefficients *Y*-intercept (0.025) and *X*_1_ (0.001). The corresponding values were less than < 0.05, hence an affirmation that the two factors are statistically significant. The coefficient β1 is 69.4032 and not 0; hence, it is statistically significant for model 1. The *Y*-intercept *t* statistic was tested and *t* = 2.31 and *X*_1_ = 3.44 (*p* < 0.05); therefore, they are statistically significant. These analyses confirm that, as an end outcome, the leadership development training had a substantial and confirmed impact on health system performance indicators based on the selected priority projects. This key finding is further supported by our hypothesis with a confidence level of *p* < 0.05.

### Multiple Regression Analysis

The association between the independent and independent variables could not exclusively be explained. Thus, model 2 introduced an additional independent variable baseline health system performance indicator before the training. Hence, from model 1, a multiple regression model was constructed to explain further the relationship between the independent variables and dependent variables, as described below. Multiple regression analysis was carried out to determine the effect of the preparation, taking into account the baseline data for the indicators of interest before the training.

Table 5 presents a summary of the regression results for model 2.

$$\begin{array}{l} \gamma =c+\beta_{1}X_{1}+\beta_{2}X_{2}+e_{t}\\ \text{where}\;\gamma =\text{health system performance}\;(\text{dependent variable}),\\ X_{1}=\text{health system performance indicators at baseline}\\ (\text{independent variable}),\\ X_{2}=\;1\;\text{for trained and}\;0\;\text{for non}-\text{trained}\\ (\text{independent variables}).\\ c=\text{constant}\\ \beta_{1}\;\text{and}\;\beta_{2\;}=\text{regression coefficients}\\ e_{t}=\text{error term} \end{array}$$

**Table 5:** Regression statistics.

Multiple (*R*)	0.9163					
*R*^2^	0.84					
Adjusted *R*^2^	0.84					
Standard Error	35.1012					
Observations	62.0000					
ANOVA						
	***df***	***SS***	***MS***	***F statistics***	***F Significance***	
Regression	2.0000	380,500.19	190,250.10	154.4124	0.0000	
Residual	59.0000	72,693.37	1,232.09			
Total	61.0000	453,193.57				
	***Coefficients***	***Standard error***	***t-Stat***	***P-value***	***Lower 95%***	***Upper 95%***
Intercept	2.4082	6.5948	0.3652	0.7163	(10.7879)	15.6044
Training/o training	50.0897	8.9996	5.5658	0.0000	32.0816	68.0978
Pretest	1.1057	0.0702	15.7553	0.0000	0.9652	1.2461

*Source: Survey data 2018*.

The variance analysis is estimated in terms of *F* statistic high accuracy of model 2, where *F* significance (0.00) is < 0.05. The *p*-value for the independent variable (*X*-intercept) *X*_1_ is .00, and therefore, they are statistically significant. *R*^2^ = 0.84 and close to 1.0; this is an indication of the high variability of the variables *X*_1_ and *X*_2_. The coefficients β1 (50.08) and β2 (1.10) are significant, meaning the two independent variables *X*_1_ and *X*_2_ influenced the dependent variable as confirmed by the confidence interval of *p* < 0.05. Model 2 demonstrated multiple *R* (0.91) and *R*^2^ (0.84), indicating that the leadership training together with the baseline results prior to the training had a considerable effect on the endline after the training and is confirmed by 84% of the variance in the health system mean indicator of the trained managers. Results reinforced the proposition that the leadership development of the health workforce positively affects the health system performance indicators based on the health system performance improvement project selected and implemented by health managers during the training results. The two models demonstrated the positive impact of the leadership program on selected project indicators because the outcome indicators scale means of the trained teams are higher than those for the non-trained managers.

### Impact Analysis

The impact is defined as “the difference in outcome between what was observed with the treatment and what would have been observed in the absence of the treatment” (39). To estimate the impact of the training, the following stepwise calculation was undertaken: (a) means and mean differences and (b) use of regression method to estimate DID structural model below by Krueger and Card (38) and Larson and Hutchinson (39). Measuring impact using means and mean differences, in other words, the impact is the DiD as represented in the equation and figure below.

$$\begin{array}{l} \left[\text{Impact}\right]\text{=}\left[\begin{array}{c} \text{Mean difference for treatment}\\ \text{ between posttest and pretest} \end{array}\right]\\ -\;\left[\begin{array}{c} \text{Mean difference for control}\\ \text{ between posttest and pretest} \end{array}\right]\\ =[(\text{89}.\text{91-30}.\text{73})\text{-}(\text{14}.\text{13-12}.\text{16})]\\ \text{Impact}=Y{}^{\text{TF}}(D\text{=1})\text{-}Y^{\text{CF}}(D\text{=}\;\text{0})\\ \text{where}:\\ Y^{\text{TF}}(D\text{=1})\text{is the mean averages of observed outcome for}\\ \text{intervention group}\\ Y^{\text{CF}}(D\text{=0})\text{is the mean averages of counterfactual outcome}\\ \text{ for non-intervention}\\ \text{Impact=}(Y^{\text{TF}}(\text{1})\text{-}Y^{\text{CF}}(\text{0})\;\geq \text{1}\\ Y^{\text{TF}}(\text{1})\text{=}\;\text{59}.\text{18}\\ Y^{\text{CF}}(\text{0})\text{=}\;\text{1}.\text{96}\\ \text{Impact=57}.\text{2},\text{therefore}\;\geq \text{1} \end{array}$$

### Answering the Research Questions

The aim of the study was to assess the efficacy and effect of leadership development training on the health system performance indicators through the implementation of institutional improvement priority projects. The first research question explored whether the training enabled participants to prioritize and successfully implemented the priority improvement projects. The pretest and posttest means for the six health system pillar indicator measures of the trained manager were higher compared to the non-trained managers. The *t*-test results revealed that training had a positive effect on the six HS pillar measurements in that posttest scales indicated a substantial variance between the treatment and control group for the three HS pillars (service delivery, LMG, and information) of 0.05 (*p* < 0.05). The second research question aimed to investigate whether the leadership development training had any impact on the health system performance indicators addressed by a trained team of managers. Linear regression analysis confirmed the improvement of HS indicator scales; hence, we can substantiate leadership training contributed positively by improving the efficiency of the health care system performance indicators through the implementation of priority challenge projects.

## Discussion

Leadership and governance building block is one of the health system building blocks widely recognized as an important determinant of strengthening national health systems and is at the core of achieving the health-related goals. Thus, leadership development training as an organizational practice has come to be recognized as the most common human resource strategy and solution for improving performance (40). Nevertheless, there is a limited body of research, particularly in regard to health care leadership capacity development and its impact in a low-income setting in sub-Saharan Africa, including Kenya. The study objective was to assess the effectiveness and impact of leadership development training on the health system performance indicators through the implementation of institutional improvement priority projects. The results also show that sustained development of leadership competencies of health workers results in improved health system performance in terms of service delivery. This finding supports previous findings from other studies (15, 17, 41–43).

The findings further revealed that the selected counties' institutional improvement projects were, in the order of priority, focused on (a) service delivery, (b) information, (c) leadership and governance, (d) health workforce, (e) medical products, and (f) finance. These emerging dynamics could be explained by the fact that teams prioritized challenges that were only within their sphere of influence and control. Even though the majority of respondents reported that human resources for health was one of their major challenges, they, however, opted for other projects based on their ability to influence and champion change. It is interesting to note that despite high reporting on health workforce as a major challenge in many institutions across all sectors in Kenya, only four teams prioritized and implemented projects focusing on human resources for health. One reason reported as to why there were few projects focusing on human resources for health was the trained managers' perception that health workforce-related challenges were way beyond their influence and control. Projects focusing on Human Resource for Health (HRH) development were also the least successful in terms of implementation. In fact, the pretest and posttest indicator scale for the human resource pillar revealed that there was no statistical difference between the treatment and control group.

Consistent with Willis-Shattuck et al.'s (44) research findings, despite the significant emphasis on human resource capacity building through training as a resolution to human resource challenges, the study participants emphasized the inadequate human resources for health as a binding constraint to the improvement of health system performance, especially in the current devolved health system in Kenya. Majority of respondents who reported that health care human resource was a major challenge were from the public and faith-based health facilities. Participants from public facilities cited the main challenges to be political in nature and limited resources to meet the public's needs and implement a new project. The private sector facilities were more concerned about the actualization of projects from paper to reality while maintaining highly qualified staff.

Endemic strikes by health care workers public health facilities were characterized by constant health worker strikes due to poor working conditions, staff shortage, and low salaries. Additionally, high staff turnover and poor retention were reported in the public and faith-based facilities as a major impediment to the implementation of the projects. The comment was evidently reflected by a couple of exciting management systems improvement projects such as automation of hospital patient and procurement systems, which are now performing at 50% capacity and in some department 0% due to high staff turnover. It was evident that some projects were not sustained as a result of championing team members, information technology team, or nurses exiting the institutions. Our participant noted the following: The devolved health system encouraged a lot of the health workforce transfers from one county to another, therefore creating gaps in some facilities. Additionally, the first phase of devolution ensured that all necessary resources were prioritized and timely; however, many counties were not able to sustain the promise, resulting in decline in performance. Some studies have suggested the need for development and implementation of appropriate policies toward retention of staff in the public sector, including both financial and non-financial incentives (15, 45).

Only 1 out of 39 priority projects were on health financing and in faith-based institutions. The LeHHO alumni alluded to the fact that some of the challenges faced by health care managers under the devolved health sector such as disparities in salaries, poor pay, lack of job security, inadequate medical supplies, and staff in health facilities were attributed to inadequate funding. Even though these challenges were common among the public health teams, the teams chose to prioritize other challenges they felt competent enough to champion. The research results for the leadership program (LeHHO) proved the leadership development training to be effective in that it was able to achieve its intended purpose, which is “to equip health care managers with knowledge, skills and attitude, which enables them to solve health care challenges.” The positive changes observed, such as attitude shift, prioritization, and implementation of learned knowledge through practice, were attributed to the leadership training. These factors are consistent with the existing leadership development literature by Kwamie et al. (41), Mansour et al. (42), Peterson et al. (17), Seims et al. (15), and West et al. (46) as evidenced by a very high percentage (92.3%) of attained “DMR” for the priority institutional improvement projects. Pretest and posttest *t*-test for the control group confirmed statistical difference for the service delivery, LMG, and information priority projects' indicators across the public, private, and faith-based health facilities.

Trained managers' outcome indicator means for all the six HS pillar indicator scales revealed significant positive improvement compared to the non-trained indicator means. Additionally, linear regression analysis revealed that those who were trained attained significant positive health system performance indicators than those who were untrained. The regression method to estimate DID structural model used to calculate the “fact” and “counterfactual” revealed that training had a positive impact on the intended outcome with impact value ≥1. Thus, the study supports both hypotheses that trained health care management teams had a significant difference in the implementation status of priority projects and, hence, had a significant impact on health system performance indicators compared with non-trained managers. As a result, the program is deemed to be achieving its intended purpose, which is to equip leaders with knowledge, skills, and practice to improve health system performance under the devolved system of government. The results show that the sustained development of leadership competencies of health workers results in improved health system performance in terms of service delivery. The results support previous findings from other studies on leadership development in sub-Saharan Africa (17, 32, 41–43, 47). The findings therefore suggest that sub-Saharan countries such as Kenya can improve their health system performance by strengthening the health system pillars through the application of leadership development practices to real work environment setting as team projects.

## Conclusion

This study sought to assess the impact of leadership development training on the health system performance indicators through the implementation of institutional improvement priority projects. The findings revealed that the trained managers achieved highly significant desired measurable results than the non-trained managers with a DID of 57.2. The findings support the presupposed hypotheses that application of leadership and governance practices centered on projects and team coaching have a beneficial impact on success in the health sector. The study, from a practical point of view, deliberated on integrated challenge-based driven methods to boost the transmission of newly learned leadership skill and knowledge through practice. The findings are important in providing guidance on innovative learning approaches that triggers immediate knowledge transfer by solving pressing challenges in the health sector. Also, the results recommend the need for leadership development among the health workers as a strategy of strengthening the other five WHO pillars of the health system (information, financing, human resources, medicines, technology, and service delivery) for quality improvement of sustainable health systems.

### Research Contributions

Published research focusing on the application of leadership practices through project implementation during the training is a relatively recent and still underrepresented topic in leadership development training programs. This study contributes to the growing body on training evaluation research and training and development literature. The study also contributes to the growing volume research employing the concept of experiment design in leadership development programs.

### Research Limitation

Although this study was carefully designed to reach its aims, we identified several limitations. First, the data sources were entirely from the Strathmore University LeHHO program alumni. This limits the findings to the program and the respective health facilities, and henceforth should be generalized with caution. Second, being an experiment, the treatment and control groups were purposive; hence, there exists potential bias. However, the two groups are governed by the same county strategic plan. Third, the study does not seek to demonstrate causation, but how leadership training could have positively contributed to improved health performance. The study looked at the improvement of health system performance through the implemented institutional improvement priority projects as a catalyst chosen by institutional teams as informed by the county or institutional strategic plans. The project served as a knowledge transfer and skill acquisition practice. Even with these limitations, the findings can be instructive in other settings and potentially valuable to support the implementation of dynamic health system strategic plans in a resource-scarce context.

## Data Availability Statement

The raw data supporting the conclusions of this article will be made available by the authors, without undue reservation.

## Ethics Statement

The study involved human participants and the ethical approval for the study was provided by the Strathmore University Ethical Review Committees (SU-IRB 0243/18) and the permit was obtained from the National Commission for Science, Technology and Innovation (NACOSTI/P/18/21001/23609). The participants provided written informed consent to participate in this study.

## Author Contributions

TC, GK, and JR conceptualized the study. TC collected the data. TC and JO analyzed and interpreted the data. TC wrote the first draft. All authors critically reviewed and approved the final manuscript.

## Conflict of Interest

The authors declare that the research was conducted in the absence of any commercial or financial relationships that could be construed as a potential conflict of interest.

## Abbreviations

LMG: Leadership, Management, and Governance
LEHHO: Leading High-Performing Healthcare Organizations
HS: Health Services
LDP: Leadership Development Program
DMR: Desired Measurable Result
SBS: Strathmore Business School
USAID: US Agency for International Development
MSH: Management Sciences for Health
SPSS: Statistical Package for Social Scientists.
